# Author Correction: Combinatorial immunotherapies overcome MYC-driven immune evasion in triple negative breast cancer

**DOI:** 10.1038/s41467-022-34570-5

**Published:** 2022-11-21

**Authors:** Joyce V. Lee, Filomena Housley, Christina Yau, Rachel Nakagawa, Juliane Winkler, Johanna M. Anttila, Pauliina M. Munne, Mariel Savelius, Kathleen E. Houlahan, Daniel Van de Mark, Golzar Hemmati, Grace A. Hernandez, Yibing Zhang, Susan Samson, Carole Baas, Marleen Kok, Laura J. Esserman, Laura J. van ‘t Veer, Hope S. Rugo, Christina Curtis, Juha Klefström, Mehrdad Matloubian, Andrei Goga

**Affiliations:** 1 Department of Cell and Tissue Biology, University of California, San Francisco, California USA; 2 Helen Diller Family Comprehensive Cancer Center, University of California, San Francisco, California USA; 3 Cancer and Developmental Therapeutics Program, Buck Institute for Research on Aging, Novato, California USA; 4 Departments of Pathology and Laboratory Medicine, University of California, San Francisco, California USA; 5 Finnish Cancer Institute, FICAN South Helsinki University Hospital & Translational Cancer Medicine, Medical Faculty, University of Helsinki, Helsinki, Finland; 6 Stanford Cancer Institute, Stanford University School of Medicine, Stanford, California USA; 7 Breast Science Advocacy Core, UCSF Breast Oncology Program, San Francisco, California USA; 8 Alamo Breast Cancer Foundation, San Antonio, Texas USA; 9 Department of Medical Oncology, Netherlands Cancer Institute, Amsterdam, The Netherlands; 10 Department of Tumor Biology & Immunology, Netherlands Cancer Institute, Amsterdam, The Netherlands; 11 Department of Surgery, University of California, San Francisco, California USA; 12 Department of Radiology, University of California, San Francisco, California USA; 13 Department of Medicine, Division of Hematology/Oncology, University of California, San Francisco, California USA; 14 Department of Medicine, Division of Oncology, Stanford University School of Medicine, Stanford, CA USA; 15 Department of Genetics, Stanford University School of Medicine, Stanford, California USA; 16 Department of Medicine, Division of Rheumatology and Rosalind Russell/Ephraim P Engleman Rheumatology Research Center, University of California, San Francisco, California USA

**Keywords:** Breast cancer, Tumour immunology, Tumour immunology

Correction to: *Nature Communications* 10.1038/s41467-022-31238-y, published online 27 June 2022

In the study, the authors use gene expression data and clinical response from patients enrolled in the TONIC trial (NCT02499367). The TONIC trial is a two-stage trial. While the stage 2 is still ongoing, results of the first stage were previously reported [10.1038/s41591-019-0432-4] and gene expression data are available on the European Genome-phenome Archive (EGA) under accession number EGAS00001003535.

In the original manuscript, the authors have mistakenly included patients enrolled in the stage 2 of the TONIC trial in the analyses related to Fig. 1l, Fig. 2b,d, and Fig. 3c. To comply with editorial policy with respect to data availability and clinical research reporting, analyses underlying Fig. 1l, Fig. 2b,d, and Fig. 3c of the manuscript have now been revised to include only patients enrolled in the published stage 1 of the TONIC trial and whose gene expression data have been deposited in EGA with accession code EGAS00001003535. These changes do not affect the scientific conclusions of the study.

In addition, Supplementary Figure 5 has also been corrected. In this case, while all of the patients reported in the original figure were from the stage I of the TONIC trial, one of the samples is not currently available in EGA and has now been removed from the analysis.

Figures and legends have been updated accordingly. The original sentence in the Figure legend “Comparison of the MYC_BC signature (MYC_Score) between responders (*n* = 8) and non-responders (*n* = 37)” now reads “Comparison of the MYC_BC signature (MYC_Score) between responders (*n* = 7) and non-responders (*n* = 37)”.

Numbers of patients have been also updated accordingly in the related Methods section “MYC signature in TONIC”. The original sentence “RNA sequencing of TNBC metastases at trial baseline (*n* = 80), post-induction (*n* = 66), or on nivolumab (*n* = 57)” now reads “RNA sequencing of TNBC metastases at trial baseline (*n* = 53) and post-induction (*n* = 44)”.

The following statement in the Methods: “We compared the MYC signature between responders and non-responders at postinduction and on-nivolumab using a logistic regression model correcting for induction strategy” now reads “We compared the MYC signature between responders and non-responders post-induction using a logistic regression model correcting for induction strategy”.

An updated Source data file is also provided with the paper.

The authors have also requested to add Dr. Marleen Kok as a co-author. Author list, Affiliation list and Author contributions (the following sentence has been added: “MK generated the TONIC trial dataset and advised on these data”) have been updated accordingly.

These changes have been implemented in the PDF and HTML versions of the Article.

