# Supplementary material for: Combinatorial immunotherapies overcome MYC-driven immune evasion in triple negative breast cancer
Source: Nat Commun. 2022 Jun 27;13:3671. doi: 10.1038/s41467-022-31238-y (PMC9237085; doi:10.1038/s41467-022-31238-y)

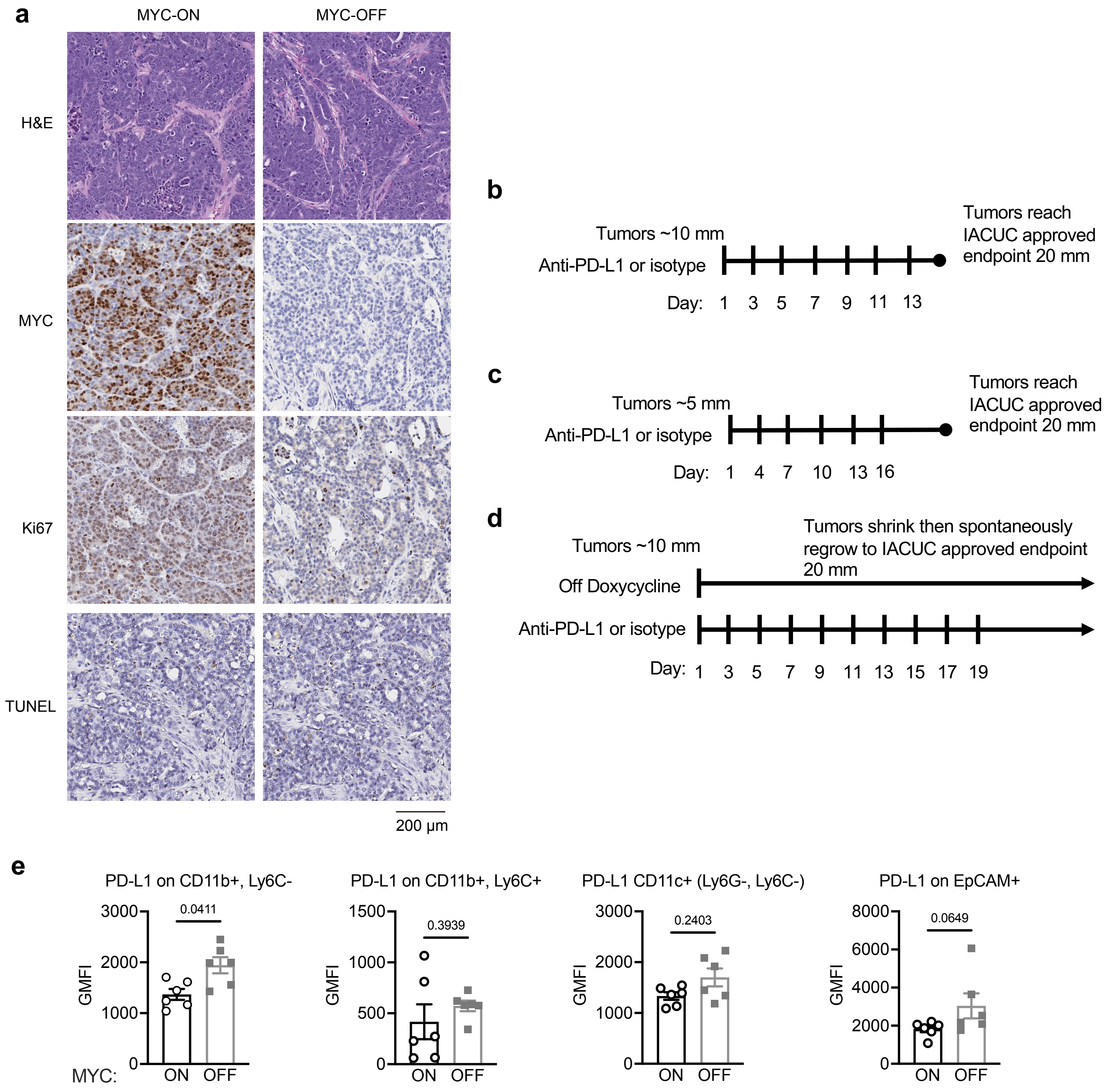

Supplementary Fig. 1: **The MYC-driven breast model for testing anti-PD-L1. (a)** Hematoxylin and Eosin staining, immunohistochemistry DAB staining for MYC, Ki67, and TUNEL in tumors from a representative animal on doxycycline chow (MYC-ON, n=3) or a representative animal 3 days off doxycycline chow (MYC-OFF, n=2). **(b-d)** Timeline for treatment related to Figure 1: Anti-PD-L1 or isotype control antibody was administered on days marked by the tick marks. **(b)** Female FVBN mice were randomized into treatment arms once transplanted tumors reached approximately 10 mm in length (longest length in any direction). **(c)** Female C57BL/6 mice were randomized into treatment arms once injected tumors formed 5 mm in length (longest in any direction). **(d)** Female FVBN mice were taken off doxycycline and randomized into anti-PD-L1 or isotype antibody once transplanted tumors reached 10 mm in length (longest in any direction). **(e)** Flow cytometry analysis of surface PD-L1 expression on cells found in MYC-ON tumors (n=6) or MYC-OFF tumors (off doxycycline chow for 3 days) (n=6). Data points are individual animals, mean+/-SEM. Two-sided, Mann-Whitney test, p values displayed above the bar graph. Source data are provided in the Source Data file.

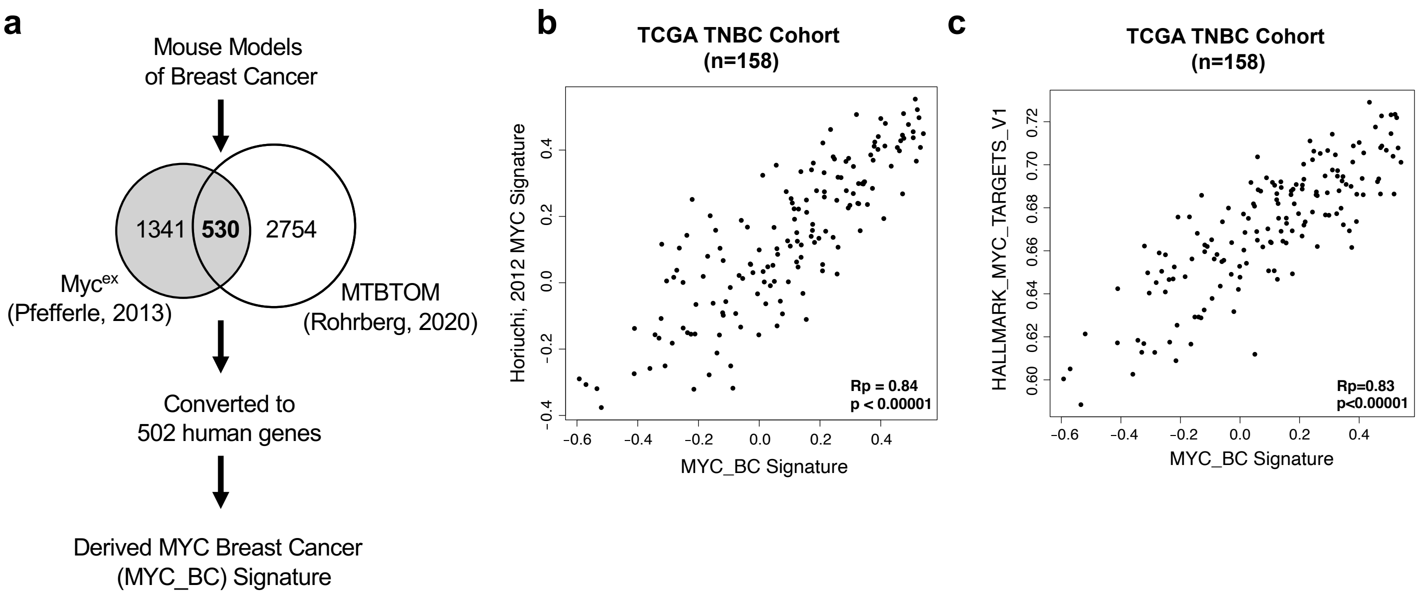

Supplementary Fig. 2: Breast tumor-derived MYC signature (MYC_BC) correlates with published MYC signatures. **(a)** Diagram depicting derivation of the MYC Breast Cancer (MYC_BC) Signature (502 genes available in Supplementary Dataset 1). **(b)** Pearson correlation was assessed between MYC_BC signature expression and 352 gene MYC signature (Horiuchi, 2012; Chandriani, 2009). **(c)** Pearson correlation was tested between MYC_BC signature expression and MSigDB MYC v1 gene set signature. TCGA TNBC cohort (n=158). Rp, Pearson's correlation coefficient, and adjusted p value, exact value shown unless below 0.00001. Source data are provided in the Source Data file.

TCGA TNBC (n=158)

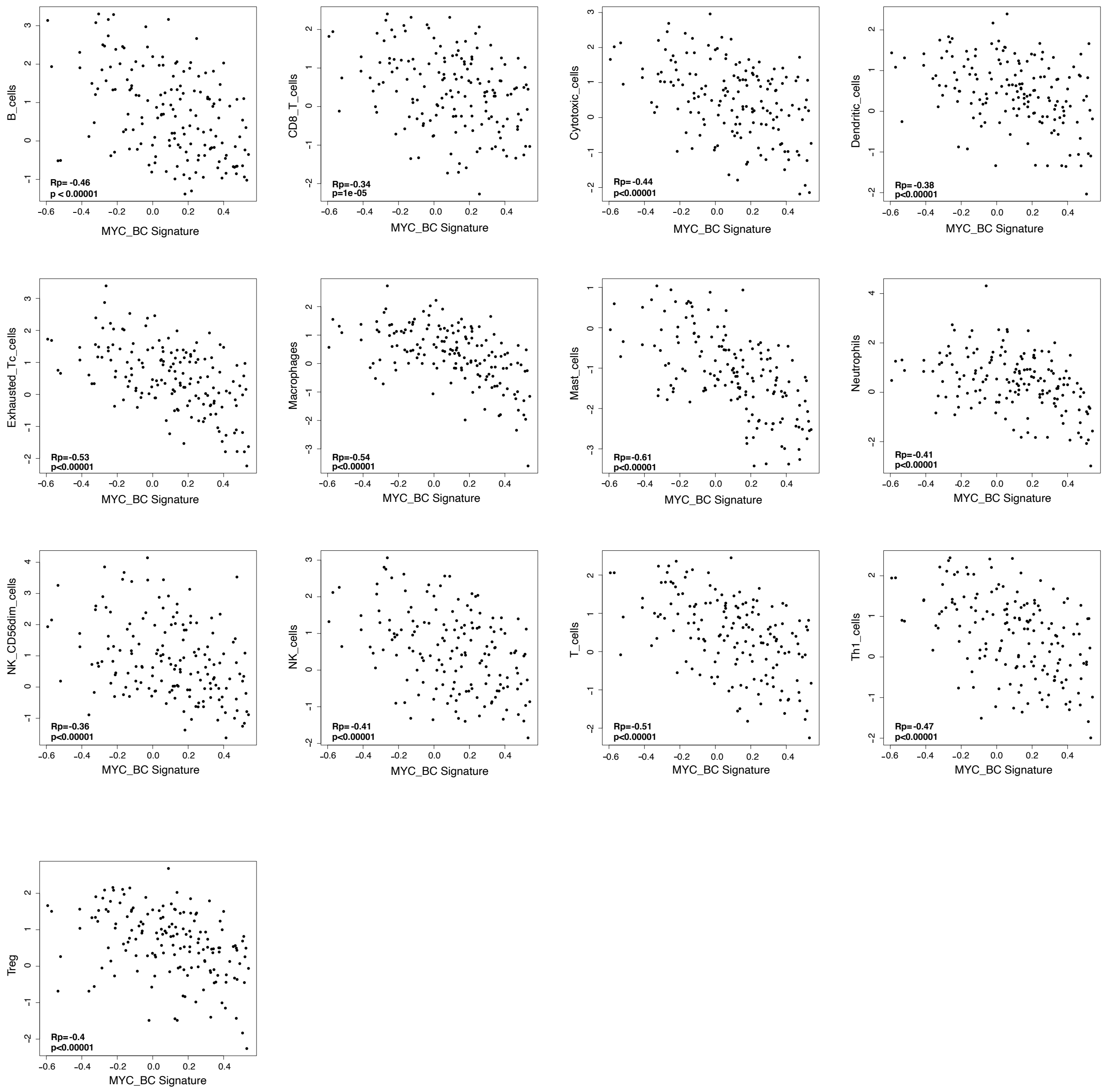

Supplementary Fig. 3: MYC_BC signature is anti-correlated with multiple immune cell signatures. Pearson correlation tested between MYC_BC signature expression and published immune signatures from Danaher, 2017. TCGA TNBC cohort (n=158). Rp, Pearson's correlation coefficient, and adjusted p value, exact value shown unless below 0.00001. Source data are provided in the Source Data file.

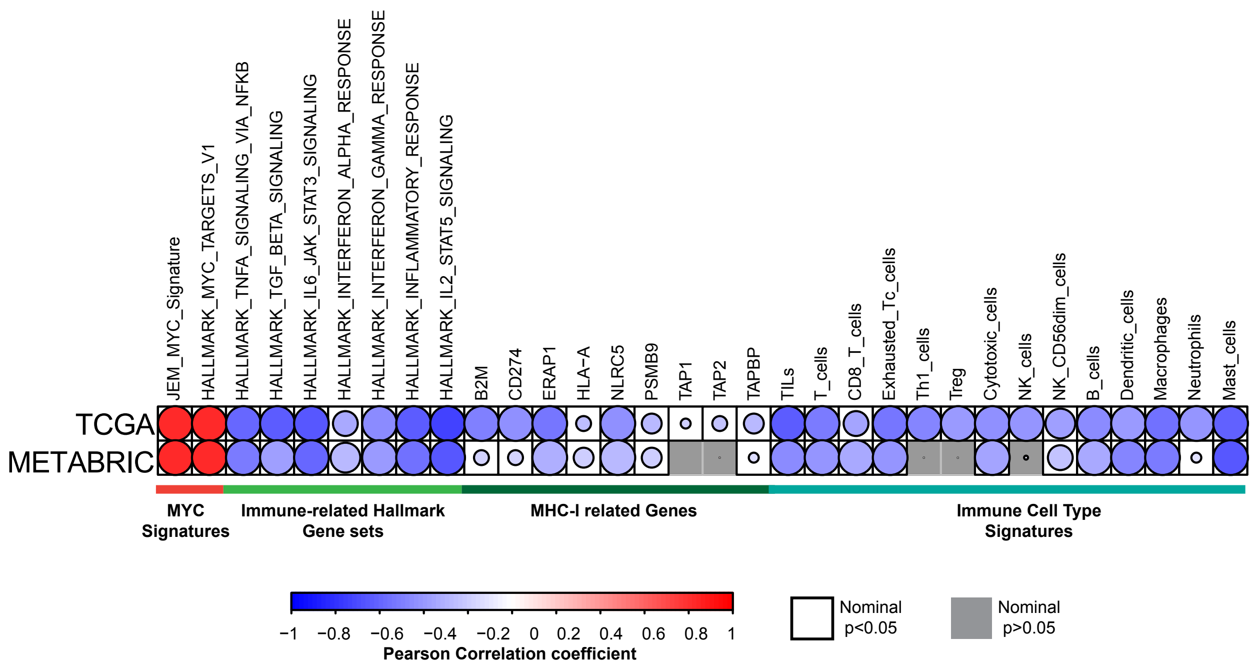

Supplementary Fig. 4: MYC_BC signature is anti-correlated with immune signatures in TCGA and METABRIC. Immune signatures and genes of interest tested for Pearson correlation with the MYC_BC signature result in similar trends in TCGA TNBC patient dataset and the METABRIC TNBC dataset. Pearson's correlation coefficient scores were computed and displayed in color for comparison. Source data are provided in the Source Data file.

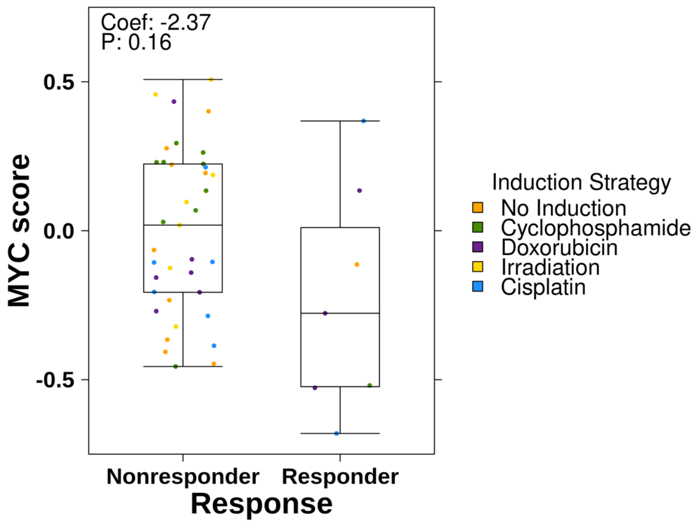

Supplementary Fig. 5: TONIC Trial. Comparison of the MYC_BC signature (MYC_Score) between responders (n=7) and non-responders (n=37), post-induction, using a logistic regression model correcting for induction strategy. Box and whiskers plot showing median, 0.25 and 0.75 quantiles, with whiskers at 1.5X interquartile range. Spearman's coefficient (Coef) and exact p value (P) displayed in upper left corner of the graph. Source data are provided at EGA (EGAS00001003535).

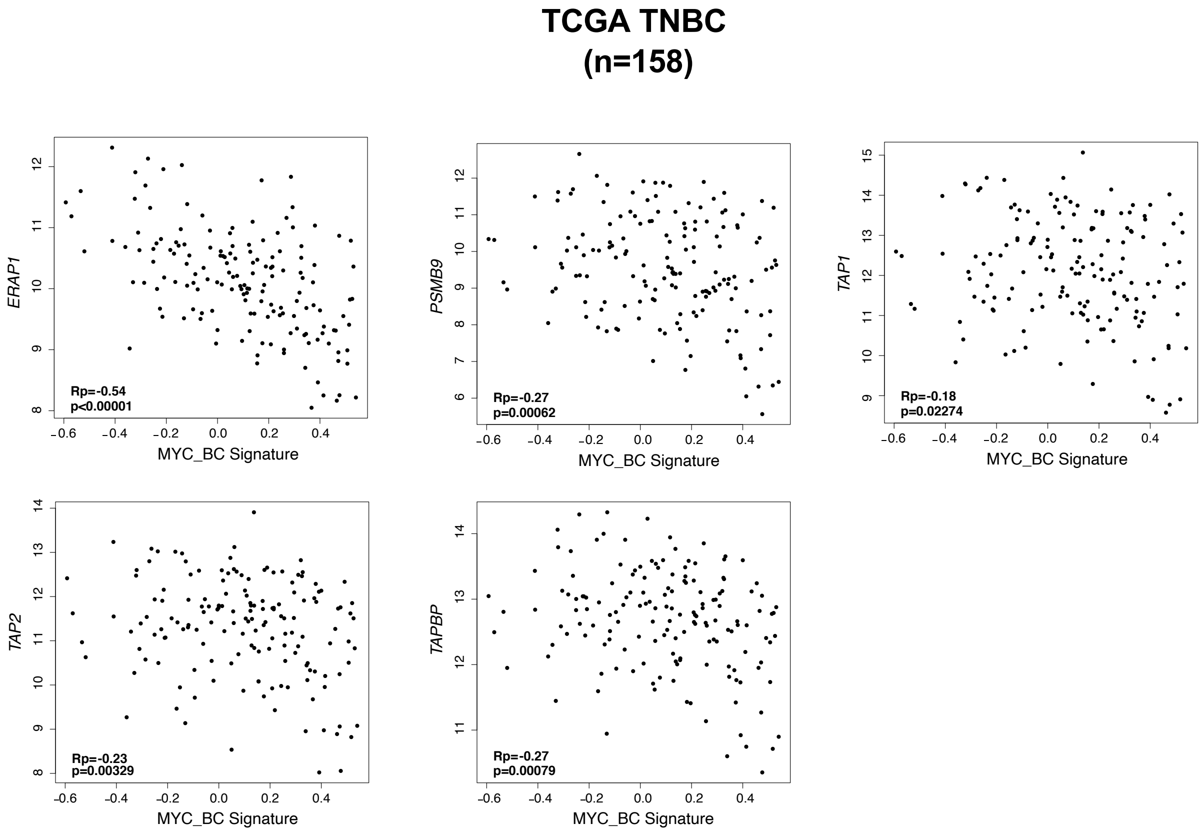

Supplementary Fig. 6: MYC_BC signature is anti-correlated with genes required for MHC-I antigen presentation. Pearson correlation (Rp) tested between MYC_BC signature and expression score for each gene in TCGA TNBC cohort (n=158). Adjusted p value, exact value shown unless below 0.00001. Source data are provided in the Source Data file.

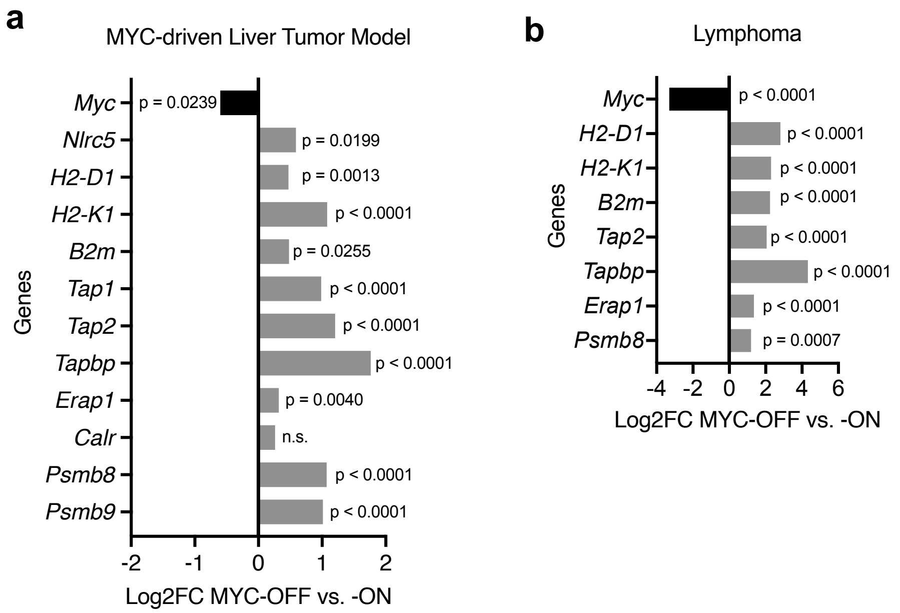

Supplementary Fig. 7: When MYC is turned off in other MYC-driven models, antigen presentation genes are upregulated. (a) RNA-seq data in a liver cancer model published by Kress et al, 2016: MYC-ON compared with MYC-OFF. Adjusted p values analyzed and reported by Kress et al. Source data available on the Gene Expression Omnibus (GEO) repository under GSE76078. **(b)** RNA-seq dataset in lymphoma cells published in Rohrberg et al 2020: MYC-OFF state compared to MYC-ON state. Adjusted p value, exact value shown unless below 0.0001, analyzed and reported in Rohrberg et al. Source data available on the GEO repository under GSE130922.

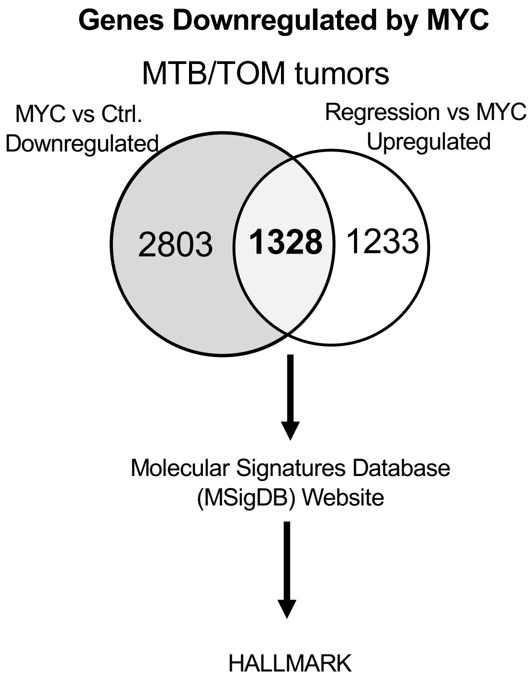

Supplementary Fig. 8: Identification of genes downregulated by MYC. The differential expression data was downloaded from Rohrberg, 2020 (GSE130922). Genes with a 2-fold change in expression and an adjusted p-value < 0.05 were selected from the published analysis. The two lists were compared using online tool Venny 2.0 (https://bioinfogp.cnb.csic.es/tools/venny/index.html) Overlapping genes were entered into GSEA/MSigDB (https://www.gsea-msigdb.org/gsea/msigdb) for evaluation of significant overlap with top 10 MSigDB HALLMARK collection.

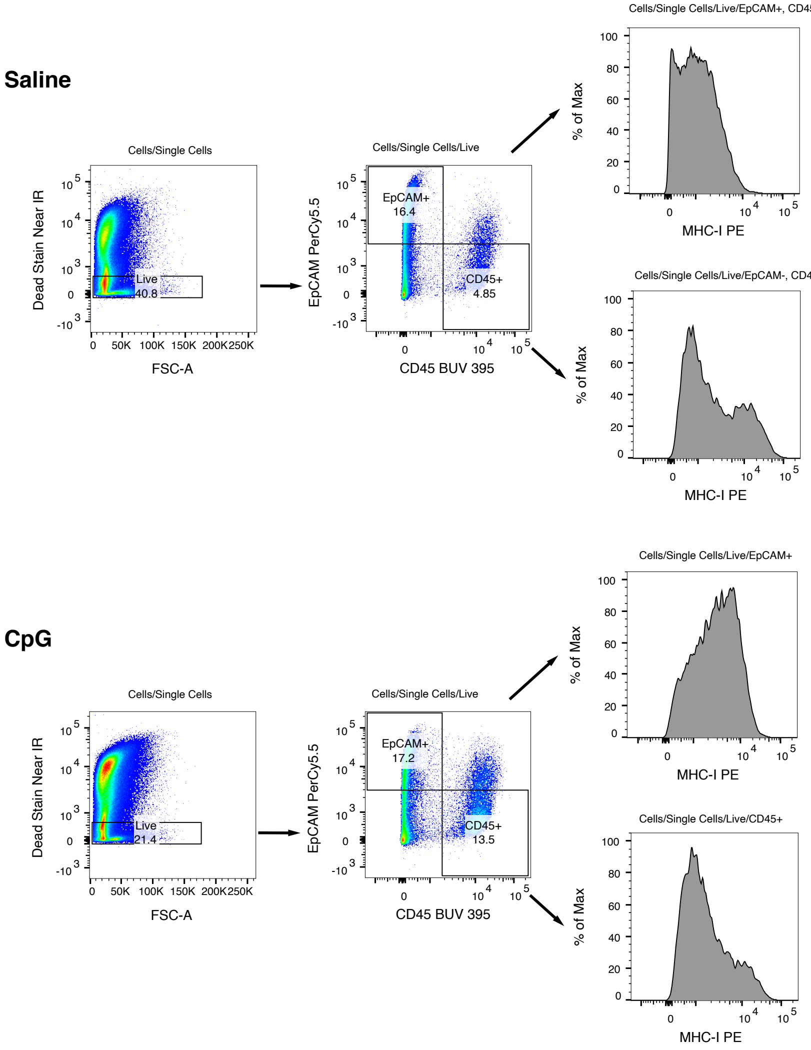

Supplementary Fig. 9. Gating strategy for MHC-I in CpG treated tumors: Cells were gated on size and granularity (SSC-A, FSC-A), Singlets (FSC-H, FSC-A), Live (negative stain), and EPCAM+, CD45- or EPCAM-, CD45+.

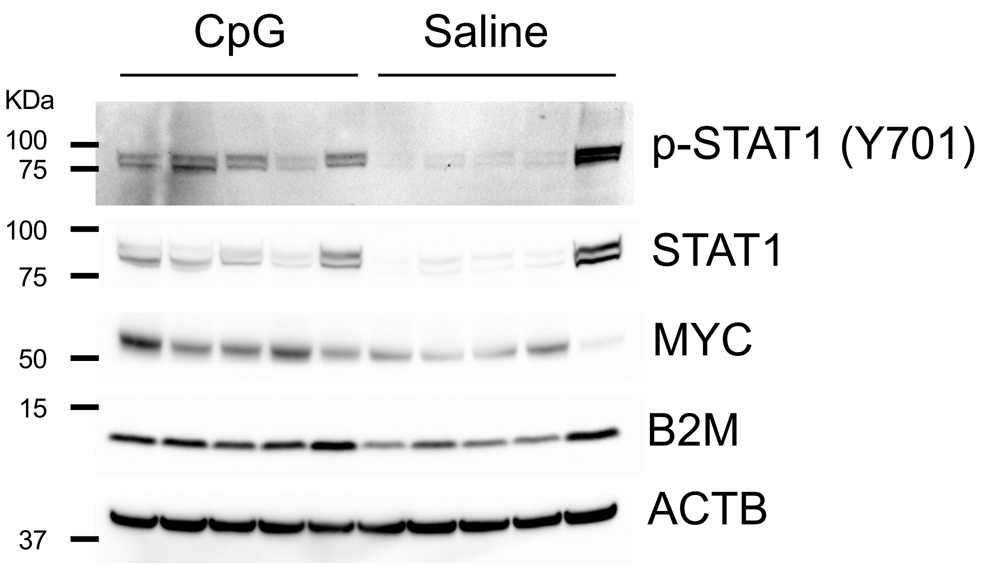

Supplementary Fig. 10. MYC expression does not change in CpG treated tumors. Western blot analysis in MTB/TOM tumors. Animals bearing MTB/TOM tumors were randomly selected to receive treatment with CpG (n=5) or saline (n=5) once tumors reached 5 mm in any direction. Tumors were harvested 48 hours post-injection. Phosphorylated-STAT1 (p-STAT1) and B2M were run on a second gel. Uncropped and unprocessed blots with the ladder displayed are provided in the Source Data file and at the end of this Supplementary Information file.

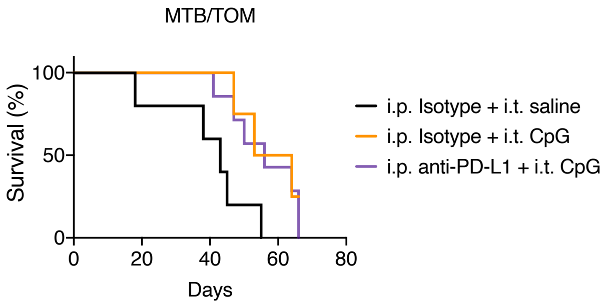

Supplementary Fig. 11: CpG+anti-PD-L1 does not extend survival beyond CpG alone. Survival analysis for MTB/TOM treated saline solution or intratumoral (i.t.) CpG and intraperitoneal (i.p.) anti-PD-L1 or isotype control antibody. Median survival was 43 days, 58.5 days and 56 days for n=5 in Isotype + saline (n=5), isotype + CpG (n=4), and anti-PD-L1+CpG (n=7), respectively. Log rank test comparing Isotype+saline to anti-PD-L1+CpG ($p = 0.0217$); Isotype+saline compared to Isotype+CpG ($p = 0.0527$); Isotype+CpG compared to anti-PD-L1+CpG ($p = 0.5819$). Source data are available in the Source Data file.

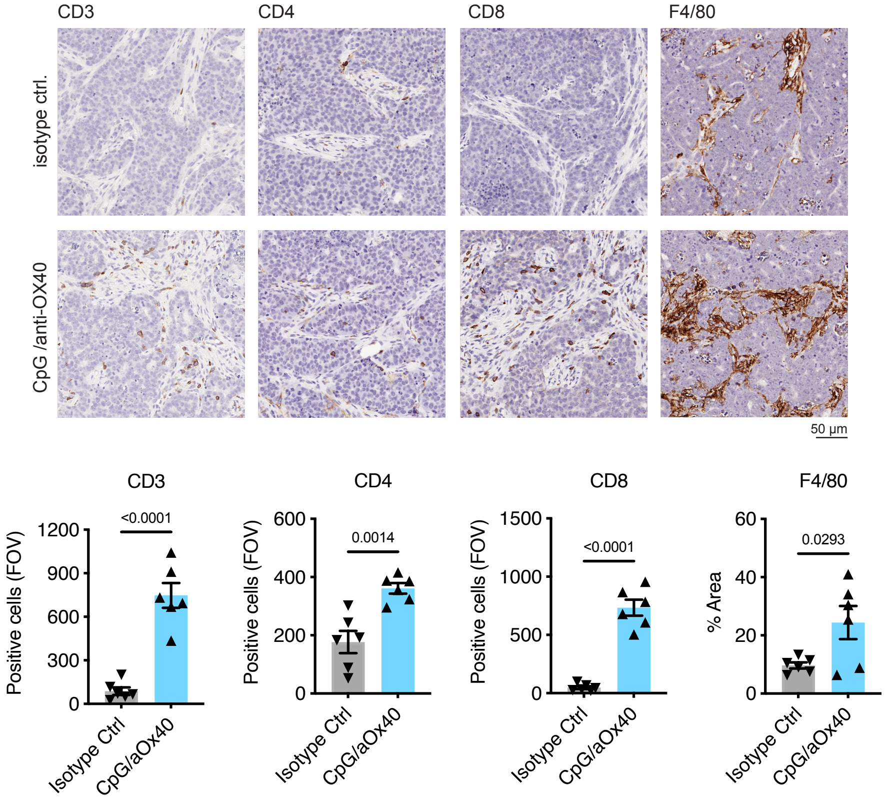

Supplementary Fig. 12: CpG/aOX40 increases T-cell infiltration. Immunohistochemistry staining for immune cells in the tumor on day 10 (top) and bar graph (below) depicting counts per field of view (FOV). Images taken at 40X. n=2 animals per group, 3 FOV analyzed per tumor, mean ± S.E.M., two-sided, unpaired t-test, exact p-values shown across the bars, unless values are less than 0.0001. Source data are available in the Source Data file.

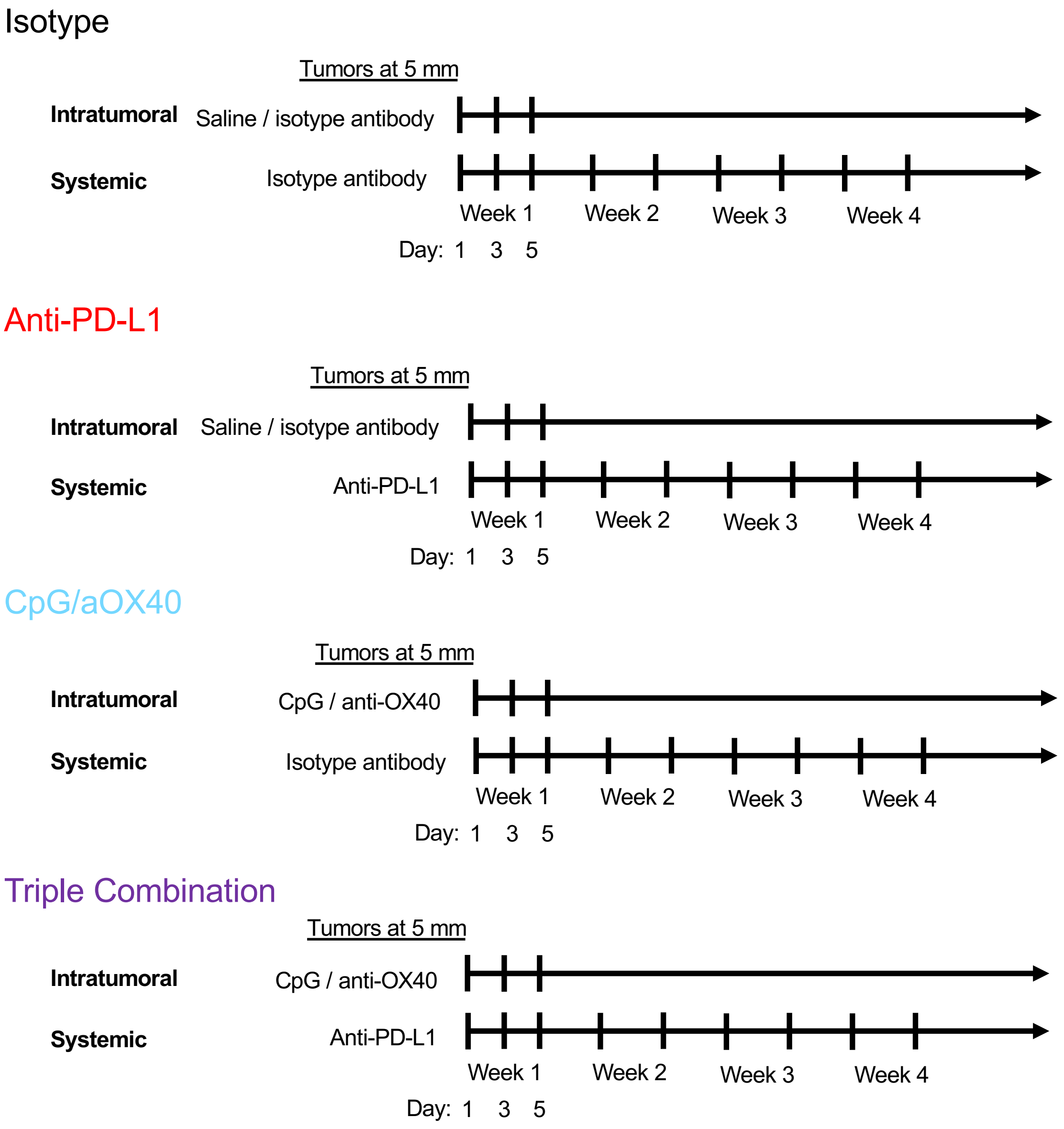

Supplementary Fig. 13: Treatment schedule related to Figure 4h, i, j, k, l. Animals were randomized into treatment arms once tumors reached 5 mm in length (longest length in any direction). All agents were administered on days 1, 3, and 5. For MTB/TOM and MC38-MYC models, animals received additional anti-PD-L1 or isotype injections twice a week in week 2, 3, and 4 (total 9 doses); WAP-MYC mice received additional anti-PD-L1 or isotype injections twice in week 2 and once in week 3, and none in week 4 (total 6 doses).

Supplementary Table 1: Pathways enriched in MYC-downregulated genes

10 Most Downregulated Hallmark Signatures

Gene Set Name	k/K	FDR q-value
HALLMARK_INTERFERON_GAMMA_RESPONSE	0.32	2.95E-46
HALLMARK_INTERFERON_ALPHA_RESPONSE	0.351	3.50E-26
HALLMARK_ALLOGRAFT_REJECTION	0.215	1.37E-23
HALLMARK_COMPLEMENT	0.165	1.22E-14
HALLMARK_INFLAMMATORY_RESPONSE	0.16	6.51E-14
HALLMARK_APOPTOSIS	0.168	2.17E-12
HALLMARK_TNFA_SIGNALING_VIA_NFKB	0.145	1.11E-11
HALLMARK_APICAL_JUNCTION	0.14	4.96E-11
HALLMARK_EPITHELIAL_MESENCHYMAL_TRANSITION	0.14	4.96E-11
HALLMARK_IL2_STAT5_SIGNALING	0.135	2.05E-10

10 Most Downregulated KEGG Signatures

Gene Set Name	k/K	FDR q-value
KEGG_ANTIGEN_PROCESSING_AND_PRESENTATION	0.18	1.13E-07
KEGG_JAK_STAT_SIGNALING_PATHWAY	0.14	1.15E-07
KEGG_FOCAL_ADHESION	0.12	1.15E-07
KEGG_FC_GAMMA_R_MEDIATED_PHAGOCYTOSIS	0.17	3.26E-07
KEGG_VASCULAR_SMOOTH_MUSCLE_CONTRACTION	0.15	7.08E-07
KEGG_COMPLEMENT_AND_COAGULATION_CASCADES	0.19	1.30E-06
KEGG_CYTOKINE_CYTOKINE_RECEPTOR_INTERACTION	0.10	1.40E-06
KEGG_TOLL_LIKE_RECEPTOR_SIGNALING_PATHWAY	0.15	3.77E-06
KEGG_TYPE_I_DIABETES_MELLITUS	0.23	4.06E-06
KEGG_ALLOGRAFT_REJECTION	0.24	9.77E-06

10 Most Downregulated GO: Biological Processes

Gene Set Name	k/K	FDR q-value
GO_REGULATION_OF_IMMUNE_SYSTEM_PROCESS	0.13	1.20E-76
GO_DEFENSE_RESPONSE	0.12	1.23E-63
GO_CELL_ACTIVATION	0.13	1.20E-59
GO_BIOLOGICAL_ADHESION	0.12	1.21E-57
GO_RESPONSE_TO_BIOTIC_STIMULUS	0.12	2.22E-56
GO_REGULATION_OF_IMMUNE_RESPONSE	0.14	1.27E-53
GO_POSITIVE_REGULATION_OF_IMMUNE_SYSTEM_PROCESS	0.13	1.28E-51
GO_IMMUNE_EFFECTOR_PROCESS	0.13	1.61E-51
GO_REGULATION_OF_RESPONSE_TO_EXTERNAL_STIMULUS	0.13	2.23E-50
GO_DEFENSE_RESPONSE_TO_OTHER_ORGANISM	0.12	1.58E-47

Supplementary Table 2: List of Antibodies used for flow cytometry experiments and immunohistochemistry (IHC) staining

Application	Name	Company	Clone/Info	Conjugate
Flow Cytometry	MHC-I (H-2Db)	eBioscience	28-14-8	PE
	CD274 (PD-L1)	Millipore	10F-9G2	PE
	CD45	BD Horizon	30-F11	BUV395
	Ep-CAM	Biolegend	G8.8	PerCPCy5.5
	FOXP3	eBioscience	FJK-16S	FITC
	Granzyme B	Biolegend	GB11	AF647
	CD8a	BD Horizon	53-6.7	BUV805
	CD4	Biolegend	RM4-5	BV605
	CD25	Biolegend	PC61	BV421
	LIVE/DEAD™ Fixable Near-IR Dead Cell Stain Kit	Invitrogen	for 633 or 635 nm excitation	
	TCR beta	Tonbo Biosciences	H57-597	PerCPCy5.5
IHC	CD3	Histowiz	SP7	
	CD8	Histowiz	4SM15	
	CD4	Histowiz	EPR19514	
	FOXP3	Histowiz	D6O8R	
	F4/80	Histowiz	BM8	
	MYC	Histowiz	Y69	
	TUNEL	Histowiz	Promega kit	
	Ki67	Histowiz	ab11580	

Supplementary Table 3: qPCR primers for mouse genes

Primer	5' to 3'
B2m Fwd	CAAATGCTGAAGAACGGGAAA
B2m Rev	ATTCAGTGTGAGCCAGGATATAG
Tapbp Fwd	TGCCAGCCTGATCTACAAATAG
Tapbp Rev	GACTCTACGCATGTGTGTTCT
Tap1 Fwd	CGT TCT CTA CCA GCT TCA GTT C
Tap1 Rev	AAG GAG TCC GGT CCA AGT AT
Tap2 Fwd	CCA GGA GAA CAG AAC ACT GAT G
Tap2 Rev	GCC ACC ACA AGG AAG AAG AA
Nlrc5 Fwd	CTCACAGGGCCAAAGATGATAG
Nlrc5 Rev	GAGGGAAATTGTGGAAGGAGAG
Psmb8 Fwd	GCT ACC CAC AGA GAC AAC TAT TC
Psmb8 Rev	GAC ATC GGA ACT CTC CAC TTT C
Psmb9 Fwd	GGT TAT GTG GAC GCA GCT TAT
Psmb9 Rev	ACT AGA GCC ATC TCG GTT CA
Actb Fwd	GAG GTA TCC TGA CCC TGA AGT A
Actb Rev	CAC ACG CAG CTC ATT GTA GA

Supplementary Table 4: qPCR primers for human genes

Primer	5’ to 3’
HLA-A Fwd	GAGGAGGAAGAGCTCAGATAGA
HLA-A Rev	GGCAGCTGTCTCACACTTTA
B2M Fwd	CCAGCGTACTCCAAAGATTCA
B2M Rev	TGGATGAAACCCAGACACATAG
NLRC5 Fwd	GACAAAGGAGTACCCTGCATTA
NLRC5 Rev	GGGTCACACTGCCACATATAA
ERAP1 Fwd	CAGGTAGGTAGAGCAAGAAGATG
ERAP1 Rev	TGAAGGAGTGGACACAGTTAAG
CALR Fwd	CGGTGACGAGGAGAAAGATAAA
CALR Rev	CTGGCCTTTGTTGCTGAAAG
PSMB8 Fwd	CGGGTGAACAAGGTGATTGA
PSMB8 Rev	CAGATAGTACAGCCTGCATTCC
PSMB9 Fwd	GCTTCACCACAGACGCTATT
PSMB9 Rev	TTGCCCAAGATGACTCGATG
MYC Fwd	GGCTCCTGGCAAAAGGTCA
MYC Rev	CTGCGTAGTTGTGCTGATGT
ACTB Fwd	GGACCTGACTGACTACCTCAT
ACTB Rev	CGTAGCACAGCTTCTCCTTAAT

Uncropped Supplementary Figure 10

Gel #1: MYC, ACTB, STAT1

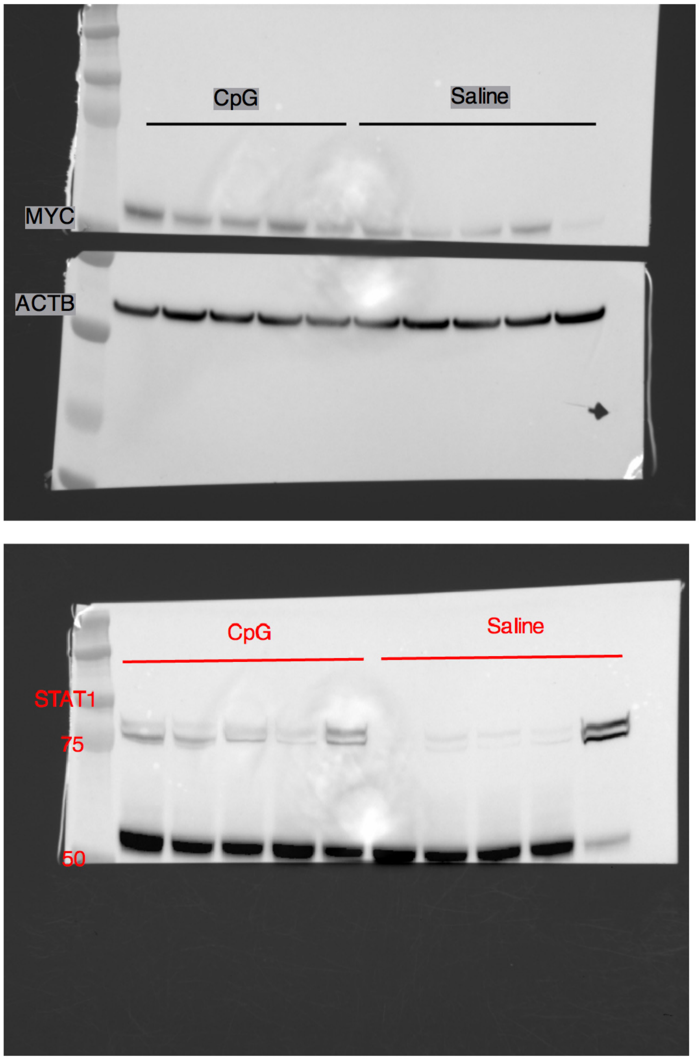

Gel #2: B2M, ACTB, pSTAT1

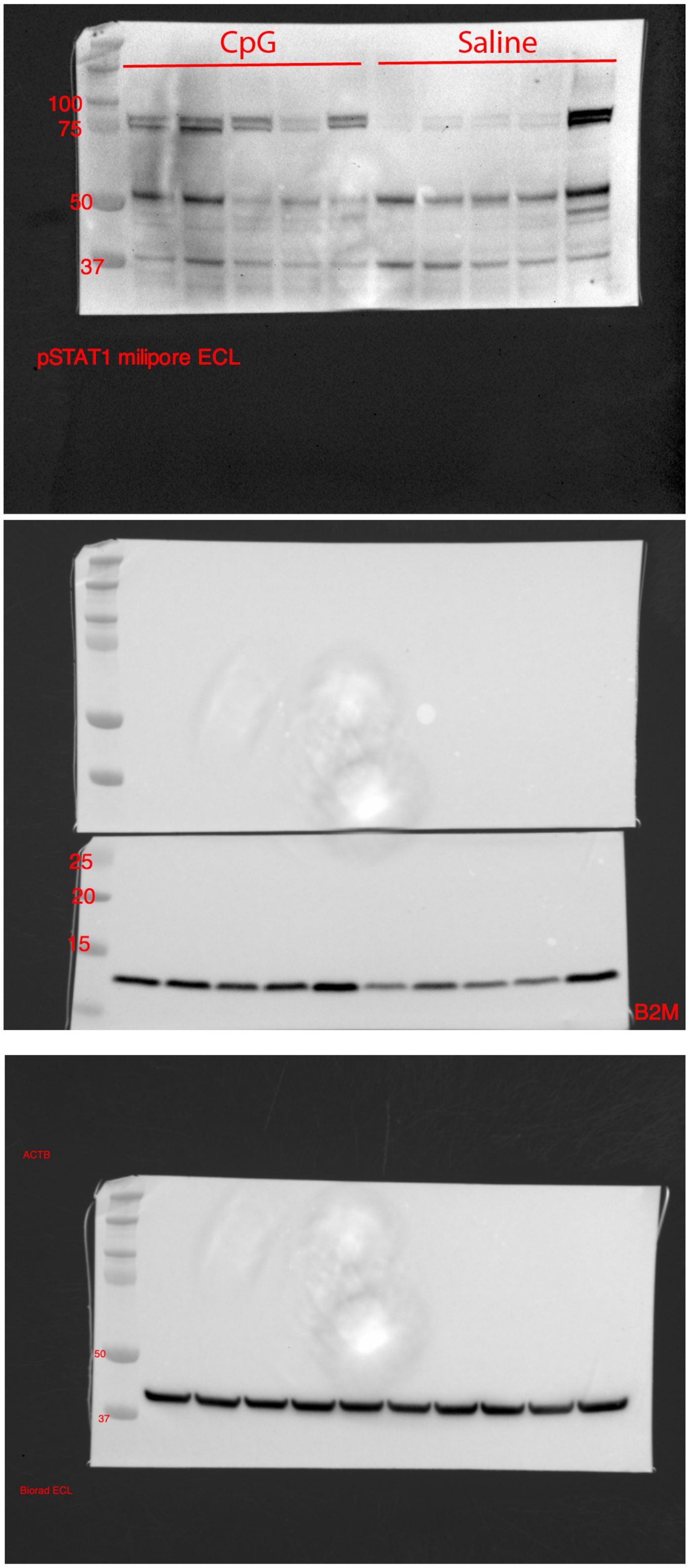

Supplement: Supplementary file 1 — Supplementary Information [file 41467_2022_31238_MOESM1_ESM.pdf]
