# Supplementary material for: Combinatorial immunotherapies overcome MYC-driven immune evasion in triple negative breast cancer
Source: Nat Commun. 2022 Jun 27;13:3671. doi: 10.1038/s41467-022-31238-y (PMC9237085; doi:10.1038/s41467-022-31238-y)
Supplement: Supplementary file 3 — Description of Additional Supplementary Files [file 41467_2022_31238_MOESM3_ESM.pdf]

Title: Supplementary Dataset 1

Description: Genes in the MYC_BC signature with the expression directionality
