# Supplementary material for: Combinatorial immunotherapies overcome MYC-driven immune evasion in triple negative breast cancer
Source: Nat Commun. 2022 Jun 27;13:3671. doi: 10.1038/s41467-022-31238-y (PMC9237085; doi:10.1038/s41467-022-31238-y)
Supplement: Supplementary file 5 — Reporting Summary [file 41467_2022_31238_MOESM5_ESM.pdf]

nature research

Corresponding author(s): Andrei Goga, Mehrdad Matloubian, Joyce V. Lee

Last updated by author(s): May 17, 2022

Reporting Summary

Nature Research wishes to improve the reproducibility of the work that we publish. This form provides structure for consistency and transparency in reporting. For further information on Nature Research policies, see our Editorial Policies and the Editorial Policy Checklist.

Statistics

For all statistical analyses, confirm that the following items are present in the figure legend, table legend, main text, or Methods section.

n/a	Confirmed	
☐	☒	The exact sample size (*n*) for each experimental group/condition, given as a discrete number and unit of measurement
☐	☒	A statement on whether measurements were taken from distinct samples or whether the same sample was measured repeatedly
☐	☒	The statistical test(s) used AND whether they are one- or two-sided<br>*Only common tests should be described solely by name; describe more complex techniques in the Methods section.*
☐	☒	A description of all covariates tested
☐	☒	A description of any assumptions or corrections, such as tests of normality and adjustment for multiple comparisons
☐	☒	A full description of the statistical parameters including central tendency (e.g. means) or other basic estimates (e.g. regression coefficient) AND variation (e.g. standard deviation) or associated estimates of uncertainty (e.g. confidence intervals)
☐	☒	For null hypothesis testing, the test statistic (e.g. *F*, *t*, *r*) with confidence intervals, effect sizes, degrees of freedom and *P* value noted<br>*Give P values as exact values whenever suitable.*
☒	☐	For Bayesian analysis, information on the choice of priors and Markov chain Monte Carlo settings
☒	☐	For hierarchical and complex designs, identification of the appropriate level for tests and full reporting of outcomes
☐	☒	Estimates of effect sizes (e.g. Cohen's *d*, Pearson's *r*), indicating how they were calculated

Our web collection on statistics for biologists contains articles on many of the points above.

Software and code

Policy information about availability of computer code

Data collection	BD FACSDiva Software (v 8.0.1) was used to collect flow cytometry data<br>QuantStudio Real Time PCR Software (v 1.7.1) was used to collect qPCR data<br>Image Lab (v 3) was used to collect western blot images
Data analysis	R (v 3.6.3) for analysis of patient data<br>Prism 9 (v 9.1.0) for statistical analysis and graphing of biological experiments<br>FlowJo (v 10.8.0) for flow cytometry analysis<br>Image J /FiJi (v 2.1.0/1.53) for immunohistochemistry quantification

For manuscripts utilizing custom algorithms or software that are central to the research but not yet described in published literature, software must be made available to editors and reviewers. We strongly encourage code deposition in a community repository (e.g. GitHub). See the Nature Research guidelines for submitting code & software for further information.

Data

Policy information about availability of data

All manuscripts must include a data availability statement. This statement should provide the following information, where applicable:

- Accession codes, unique identifiers, or web links for publicly available datasets
- A list of figures that have associated raw data
- A description of any restrictions on data availability

No new datasets were generated in this study. The RNAseq data for MYC-driven mouse models of cancer were previously analyzed and published; the data are available through Gene Expression Omnibus (GEO) repositories: GSE130922 and GSE76078. TCGA datasets analyzed in this study are publicly available through cBioPortal. The ISPY-2 datasets analyzed in this study were previously published are available from the ISPY-2 Trial but restrictions apply to the availability of these data, so are not publicly available; the data is available by request with ISPY-2 Trial (ispyadmin@ispytrials.org). The TONIC Trial and METABRIC gene expression data are available on the European Genome-phenome Archive (EGA) under accession number EGAS0001003535 and EGAS00000000083, respectively. For gene ontology, we accessed the Molecular Signatures Database website (v 6.4). Patient outcomes for IMvigor210 Trial and metastatic ccRCC are available on the Tumor Immune Dysfunction and Exclusion (TIDE) database. The remaining data are available within the Source Data file.

Field-specific reporting

Please select the one below that is the best fit for your research. If you are not sure, read the appropriate sections before making your selection.

☒ Life sciences ☐ Behavioural & social sciences ☐ Ecological, evolutionary & environmental sciences

For a reference copy of the document with all sections, see nature.com/documents/nr-reporting-summary-flat.pdf

Life sciences study design

All studies must disclose on these points even when the disclosure is negative.

Sample size	Exact sample size varied between experiments and further details is available in the figure legends. Broadly, minimum sample size for animal experiments were chosen based on power calculation estimates and additional mice were transplanted with tumors achieve the minimum number of qualifying tumors for enrollment into treatment arms. Sample size for patient samples were based on availability from published datasets. The number of patients in METABRIC and the TCGA datasets provided sufficient statistical power to detect differences, but the I-SPY2 and TONIC datasets were underpowered, as we described in the manuscript.
Data exclusions	All data from experiments and animals are reported, except in figure 2i, where one outlier was found using Prism software (using ROUT Q=1%) and removed.
Replication	Animals experiments were replicated over 2 or more cohorts with all treatment arms represented in each cohort. The data shows all cohorts combined. Cell line experiments were all replicated independently by collecting cells of distinct passages for analysis. All trends replicated.
Randomization	Animals in each cage were randomly assigned ear tags and treatment groups. Each cage contained a mixture of all treatment groups to reduce cage-to-cage bias. For cell line experiments, samples were randomized into treatment groups.
Blinding	Investigators were not blinded to group allocation during data collection or data analysis because the investigator performing the experiment collected and labeled the samples. Blinding was not necessary for computational analysis.

Reporting for specific materials, systems and methods

We require information from authors about some types of materials, experimental systems and methods used in many studies. Here, indicate whether each material, system or method listed is relevant to your study. If you are not sure if a list item applies to your research, read the appropriate section before selecting a response.

Materials & experimental systems

n/a	Involved in the study	
☐	☒	Antibodies
☐	☒	Eukaryotic cell lines
☒	☐	Palaeontology and archaeology
☐	☒	Animals and other organisms
☐	☒	Human research participants
☒	☐	Clinical data
☒	☐	Dual use research of concern

Methods

n/a	Involved in the study	
☒	☐	ChIP-seq
☐	☒	Flow cytometry
☒	☐	MRI-based neuroimaging

Antibodies

Antibodies used	Flow Cytometry (all at 1:100 in flow cytometry buffer):<br>MHC-I (H-2Db) PE eBioscience clone 28-14-8 Catalog # 12-5999-82<br>PD-L1 PE Millipore clone 10F-9G2 Catalog # MABF404<br>CD45 BUV395 BD Horizon clone 30-F11 Catalog # 564279<br>Ep-CAM PerCPCy5.5 Biolegend clone G8.8 Catalog # 118220<br>FOXP3 FITC eBioscience clone FJK-16S Catalog # 11-5773-82<br>Granzyme B AF647 Biolegend clone GB11 Catalog # 515406<br>CD8 BUV805 BD Horizon clone 53-6.7 Catalog # 612898<br>CD4 BV605 Biolegend clone RM4-5 Catalog # 100547<br>CD25 BV421 Biolegend clone PC61 Catalog # 562606<br>TCR beta PerCPCy5.5 Tonbo Biosciences clone H57-597 Catalog # 65-5961-U100<br><br>Used aliquots at 1:1000 in PBS<br>LIVE/DEAD™ Fixable Near-IR Dead Cell Stain Kit, for 633 or 635 nm excitation Invitrogen Catalog #L10119<br><br>Immunohistochemistry [performed by Histowiz Inc (Brooklyn, NY, USA)]:<br>MYC clone Y69 Abcam Catalog # ab32072<br>CD3 clone SP7 Abcam Catalog #ab16669<br>CD4 clone EPR19514 Abcam Catalog #ab183685<br>CD8 clone 4SM15 eBioscience Catalog #14-0808-82<br>FOXP3 clone D6O8R Cell Signaling Technology Catalog #CST12653<br>F4/80 clone BM8 eBioscience Catalog #14-4801-82<br>Ki67 Abcam Catalog #ab15580<br>TUNEL (Promega)<br><br>Western Blot: Anti-ß-actin (1:10,000, sc-47778 HRP, Santa Cruz Biotechnology), anti-c-MYC (1:1,000, clone Y69, ab32072, Abcam), anti-STAT1 (1:1,000, #9172, Cell Signaling Technology), Anti-Rabbit IgG (1:10,000, #7074, Cell Signaling Technology).<br><br>Anti-PD-L1 (Bio X Cell #BE0101) for mouse<br>Anti-OX40 (Bio X Cell #BE0031) for mouse<br>Control antibodies (Bio X cell #BE0290, #BE0090)
Validation	Antibodies for flow cytometry were validated by the manufacturers for mouse (see antibody profiles on manufacturer's websites for validation information). Histowiz Inc validated antibodies in mouse tissues (posted on their website) and used positive and negative controls for each IHC stain. Western blot antibodies were validated using positive and negative control samples. BioXCell validates their antibodies using a library of recombinant proteins (see their website for more information).

Eukaryotic cell lines

Policy information about cell lines

Cell line source(s)	MCF10A cells were previously published by the Goga lab (Martins, M. M. et al. Linking tumor mutations to drug responses via a quantitative chemical-genetic interaction map. Cancer Discov 5, 154-167, doi:10.1158/2159-8290.CD-14-0552 (2015)) and sourced from the Goga Lab. MTB/TOM cell line was previously published by the Goga lab (Rohrberg, J. et al. MYC Dysregulates Mitosis, Revealing Cancer Vulnerabilities and sourced from the Goga Lab. Cell Rep 30, 3368-3382 e3367, doi:10.1016/j.celrep.2020.02.041 (2020)). MC38 cells were a gift from the Matthew Spitzer Lab which they purchased commercially.
Authentication	No cell line authentication used. Cells were routinely checked for MYC expression by western blot.
Mycoplasma contamination	Cell lines continuously tested negative for mycoplasma by PCR.
Commonly misidentified lines<br>(See ICLAC register)	This study did not use commonly misidentified lines.

Animals and other organisms

Policy information about studies involving animals; ARRIVE guidelines recommended for reporting animal research

Laboratory animals	We purchased female 4-week-old FVB/NJ and female 6-week-old C57BL/6J from Jackson Laboratories for studies conducted at the University of California San Francisco facilities. 4-week-old female FVB/NRj mice were purchased from Janvier for studies conducted at the University of Helsinki. All animals were housed in pathogen-free facilities (SPF), under standard conditions, in ventilated cages with enrichment, at 72 degrees Fahrenheit, 60% humidity, 12 hour light/ 12 hour dark cycles, and standard water and diet.
Wild animals	The study did not have wild animals.
Field-collected samples	The study did not involve field-collected samples.

Ethics oversight	Experimental procedures conducted at the University of California, San Francisco were approved by IACUC under AN184330-01. All animals in Finland were covered by a license (ESAVI-2010-05551_Ym-23, KEK19-002) approved by the National Animal Experiment Board of Finland (Eläinkoelautakunta, ELLA).

Note that full information on the approval of the study protocol must also be provided in the manuscript.

Human research participants

Policy information about studies involving human research participants

Population characteristics	De-identified gene expression data from patients with triple negative breast cancer were selected for analysis in this study: From the 1100 TCGA Research Network breast samples, 158 TNBC samples were selected based on ER and PR status by IHC and HER status by IHC or FISH (HER2-positive if either IHC or FISH is positive).<br>From METABRIC, TNBC patients were selected based expression, as described in the original METABRIC paper (Curtis, C. et al. The genomic and transcriptomic architecture of 2,000 breast tumours reveals novel subgroups. Nature 486, 346-352, doi:10.1038/nature10983 (2012)).<br>From the I-SPY2 trial, 28 TNBC patients were selected from the published study (Nanda, R. et al. Effect of Pembrolizumab Plus Neoadjuvant Chemotherapy on Pathologic Complete Response in Women With Early-Stage Breast Cancer: An Analysis of the Ongoing Phase 2 Adaptively Randomized I-SPY2 Trial. JAMA Oncol 6, 676-684, doi:10.1001/jamaoncol.2019.6650 (2020)).<br>From the TONIC trial, patients with metastatic TNBC from all treatment arms from the published study (Voorwerk, L., Slagter, M., Horlings, H.M. et al. Immune induction strategies in metastatic triple-negative breast cancer to enhance the sensitivity to PD-1 blockade: the TONIC trial. Nat Med 25, 920–928 (2019). https://doi.org/10.1038/s41591-019-0432-4) were included in our analysis.
Recruitment	No recruitment was conducted during this study.
Ethics oversight	The TCGA Ethics, Law and Policy Group established informed consent guidelines for the use of TCGA cancer genomics data. The METABRIC protocol was approved by site institutional review board. The I-SPY2 TRIAL study was registered on ClinicalTrials.gov under study number NCT01042379 and all participating sites received institutional review approval. TONIC trial was registered on ClinicalTrials.gov under study number NCT02499367 and approved by the site institutional medical ethics committee. Patients in both trials gave informed consent.

Note that full information on the approval of the study protocol must also be provided in the manuscript.

Flow Cytometry

Plots

Confirm that:

- [x] The axis labels state the marker and fluorochrome used (e.g. CD4-FITC).
- [x] The axis scales are clearly visible. Include numbers along axes only for bottom left plot of group (a 'group' is an analysis of identical markers).
- [x] All plots are contour plots with outliers or pseudocolor plots.
- [x] A numerical value for number of cells or percentage (with statistics) is provided.

Methodology

Sample preparation	Each tumor was minced with a clean blade and then dissociated in 5 mL RPMI (Gibco) containing 1 mg/mL collagenase II (Gibco) for immunophenotyping or collagenase IV (Gibco) for MHC-I, 40 µg/mL DNase (Roche), 2% heat inactivated FBS (Gibco), and 10 mM HEPES at 37 °C with constant agitation of 180 rpm for 25 minutes. Digested tissue was diluted with 30 mL of cold PBS and poured through a 70-micron nylon mesh strainer (Fisher) for tumor cell analyses or 40-micron nylon mesh strainer for immunophenotyping. Cells were pelleted at 220-300 x g and resuspended in 5 mL RBC lysis buffer (BioLegend) at room temperature. After 5 minutes, the cells were diluted with 25 mL of FACS buffer (HBSS with 1 mM EDTA and 2% heat-inactivated FBS), pelleted, and resuspended in < 3 mL of PBS for cell counts. Cells were stained with the antibody panel for 30 minutes on ice, covered. Cells were washed with PBS and stained with fixable Near-IR live/dead stain (Molecular Probes) at 1:1,000 for 15 minutes at room temperature. For FOXP3 and Granzyme B staining, cells were fixed and permeated with a transcription factor staining buffer set (Invitrogen 00-5523-00) following staining of extracellular proteins. Cells were washed and resuspended in FACS buffer for data collection on a BD Dual Fortessa, using BD FACSDiva software (v 8.0.1), and analyzed with FlowJo (v 10.8.0). For MTB/TOM cells grown in culture, the same reagents and protocols were used after cells were lifted off the plate with a cell lifter (Corning), but the RBC lysis step was omitted. All experiments were compensated with single color controls and gating was determined by full panel minus one antibody or isotype antibody controls.
Instrument	BD Dual Fortessa
Software	Data was collected using BD FACSDiva (v 8.0.1) and analyzed with FlowJo (v 10.8.0).
Cell population abundance	At least 20,000 Live events (negative for Dead stain) were analyzed for each sample.
Gating strategy	All experiments were compensated with single color controls and gating was determined by using positive and negative cell populations and full-panel-minus-one antibody or isotype antibody controls. Gating is indicated on the graphs in the figures.<br>MHC-I: All Events/Singlets/Live/EpCAM+, CD45- | Geometric Mean (MHC-I)

T-cells: All Events/Single Cells/Live/TCRb+, CD45+
CD4+ T-cells: All Events/Single Cells/Live/TCRb+, CD45+/CD8- , CD4+
CD8+ T-cells: All Events/Single Cells/Live/TCRb+, CD45+/CD8+ , CD4-
Tregs: All Events/Single Cells/Live/TCRb+, CD45+/CD8- , CD4+/FOXP3+, CD25+
Granzyme B: All Events/Single Cells/Live/TCRb+, CD45+/CD8+, CD4- | Geometric Mean (GrB)

☒ Tick this box to confirm that a figure exemplifying the gating strategy is provided in the Supplementary Information.
